# Quality-of-Life Outcomes Following Thyroid Surgery in Pediatric Patients: A Systematic Review of Physical, Emotional, and Social Dimensions

**DOI:** 10.3390/children12070891

**Published:** 2025-07-07

**Authors:** Amani N. Alansari, Mohamed Sayed Zaazouee, Safaa Najar, Alaa Ahmed Elshanbary

**Affiliations:** 1 Department of Pediatric Surgery, Hamad Medical Corporation, Doha 3050, Qatar; snajar@hamad.qa; 2 Faculty of Medicine, Al-Azhar University, Assiut 71524, Egypt; mohamedzaazouee.stu.6.44@azhar.edu.eg; 3 Faculty of Medicine, Alexandria University, Alexandria 21526, Egypt; alaa.mohamed1603@alexmed.edu.eg

**Keywords:** pediatric thyroid surgery, quality of life, systematic review, psychosocial outcomes, surgical approaches

## Abstract

**Background:** Thyroid disorders are significant endocrine conditions in pediatric populations, sometimes requiring surgical intervention. While surgical outcomes are well-documented, the broader impact on quality of life (QoL) remains insufficiently synthesized. This systematic review aimed to evaluate the effects of thyroid surgery on QoL in pediatric patients, focusing on physical, emotional, and social dimensions. **Methods:** Following the PRISMA guidelines, we searched PubMed, EMBASE, and SCOPUS from inception to January 2025. Studies reporting health-related QoL outcomes in pediatric patients undergoing thyroid surgery were included. Quality assessment was conducted using the Newcastle–Ottawa Scale. Data synthesis focused on emotional and psychological outcomes, social functioning, physical health, and surgical-approach-specific effects. **Results:** Five studies (ranging from 37 to 92 participants) met the inclusion criteria. Unilateral thyroidectomy was associated with better QoL outcomes compared to bilateral procedures, particularly in emotional and physical domains. Post-surgical anxiety significantly improved. However, male survivors reported higher levels of depression and reduced motivation. Employment status emerged as a significant factor influencing physical functioning scores. Thyroid cancer patients demonstrated better social functioning than peers with other cancers, yet they lagged behind healthy controls. Long-term follow-up highlighted ongoing challenges in physical functioning and fatigue. **Conclusions:** Thyroid surgery impacts multiple dimensions of QoL in pediatric patients, with variations depending on surgical approach and patient characteristics. These findings underscore the need for comprehensive post-operative care, including routine QoL assessments and tailored psychological support. Future research should aim to standardize assessment timing and develop targeted interventions for high-risk groups.

## 1. Introduction

Thyroid disorders are among the most common endocrine conditions affecting children and adolescents, with an estimated prevalence of 1–2% for thyroid nodules and approximately 0.02% for differentiated thyroid carcinoma in pediatric populations [1,2]. Additionally, thyroglossal cysts, which are common congenital neck masses in children, can sometimes be mistaken for thyroid-related issues, as they may present in the midline of the neck and require careful evaluation to differentiate them from thyroid pathology [3]. Graves’ disease, the most frequent cause of hyperthyroidism in children, affects about 1 in 10,000 individuals under 15 years old [4]. Surgical intervention, including total or partial thyroidectomy, is sometimes necessary, with complication rates ranging from 1 to 5% for hypoparathyroidism and 1–3% for recurrent laryngeal nerve injury [5]. While surgery is generally safe and effective, its impact extends beyond the operating room, influencing various aspects of a child’s physical, emotional, and social well-being [6]. Understanding quality of life (QoL) following thyroid surgery in pediatric patients is crucial for optimizing post-operative care and providing a more comprehensive approach to treatment.

Pediatric patients undergoing thyroid surgery face unique challenges that differ from those encountered by adults. The relationship between endocrine function, growth, and psychosocial development means that any alteration in thyroid physiology can have profound consequences [7]. Hormonal imbalances, surgical complications, and the psychological effects of a chronic condition all contribute to post-operative outcomes [8]. While some children recover smoothly, others experience long-term effects such as fatigue, voice changes, weight fluctuations, and emotional distress, which can significantly affect their daily lives [9,10]. The necessity for lifelong hormone replacement therapy, in many cases, further complicates their health pathway [11]. Therefore, evaluating QoL in this population is essential for identifying gaps in post-surgical care and improving patient-centered outcomes.

The existing literature on pediatric thyroid surgery has predominantly focused on surgical techniques, complication rates, and disease prognosis [12,13,14]. However, fewer studies have systematically examined how these procedures influence patients’ overall well-being and long-term satisfaction [15,16]. This systematic review evaluates the impact of thyroid surgery on pediatric patients’ quality of life, including physical health, emotional well-being, social adaptation, and treatment burden. Despite advances in surgery and management, individualized approaches remain limited. By synthesizing current research, this review aims to refine treatment guidelines, improve follow-up care, and enhance psychological support for better post-operative outcomes.

## 2. Materials and Methods

### 2.1. Study Design

This systematic review was conducted following the Cochrane Handbook and Preferred Reporting Items for Systematic Reviews and Meta-Analyses (PRISMA) guidelines [17,18].

### 2.2. Eligibility Criteria

Studies were included based on the following criteria: pediatric and adolescent patients who underwent thyroid surgery for differentiated thyroid carcinoma (DTC) or Graves’ disease. Eligible interventions included total thyroidectomy, hemithyroidectomy, and bilateral or unilateral thyroidectomy. Studies were considered if they compared different surgical approaches or reported health-related quality of life (HRQoL) outcomes, even in the absence of a direct comparator. Only studies that assessed HRQoL using validated tools such as the Pediatric Quality of Life Inventory (PedsQL) [19], European Organization for Research and Treatment of Cancer Quality of Life Questionnaire-Core 30 (EORTC QLQ-C30) [20], Thyroid Cancer-Specific Quality of Life Questionnaire (THYCA-QoL) [21], World Health Organization Quality of Life-100 (WHOQOL-100) [22], Child Health Questionnaire-Child Form 87 (CHQ-CF87) [23], Short Form-36 Health Survey (SF-36) [24], or Multidimensional Fatigue Inventory-20 (MFI-20) were included [25]. The review encompassed cross-sectional studies, prospective and retrospective cohort studies, and other observational designs. Articles had to be published in English and be available as peer-reviewed full-text publications. Studies were excluded if they focused solely on adult populations, did not report HRQoL outcomes, or were reviews, editorials, or case reports.

### 2.3. Information Sources, Search Strategy, and Study Selection

A comprehensive search was performed in PubMed, Embase, and Scopus from inception to January 2025. The search strategy was developed using Medical Subject Headings (MeSH) and free-text terms related to pediatric thyroid surgery and quality of life. Boolean operators (AND, OR) were applied to refine the search. The detailed search strategy for each database is provided in Table A1. The search results were imported into EndNote and deduplicated. Two independent reviewers screened the titles and abstracts against the eligibility criteria. The full texts of potentially relevant studies were retrieved and assessed for eligibility. Discrepancies were resolved by discussion or consultation with a third reviewer.

### 2.4. Data-Collection Process

A standardized data-extraction form was used to collect information on study characteristics, including the author(s), year, and country of the study; the study design and sample size; population characteristics such as age, gender, diagnosis, and surgical procedure; the HRQoL assessment tools and reported outcomes; the follow-up duration; and key findings related to emotional, social, and physical functioning. Data extraction was conducted independently by two reviewers, and any discrepancies were resolved through discussion.

### 2.5. Risk of Bias Assessment

The Newcastle–Ottawa Scale (NOS) was used to assess the quality of the included observational studies [26]. The scale evaluates three domains: selection, comparability, and outcome. Studies scoring 7–9 were considered high-quality, 4–6 were considered moderate-quality, and <4 were considered low-quality. Two independent reviewers conducted the risk of bias assessment, with disagreements resolved through consensus.

### 2.6. Data Synthesis

A qualitative synthesis was conducted, summarizing HRQoL outcomes across emotional, social, and physical domains. Due to heterogeneity in study designs, outcome measures, and assessment tools, a meta-analysis was not performed. The findings were presented narratively and in tabular format.

## 3. Results

**Figure 1:**
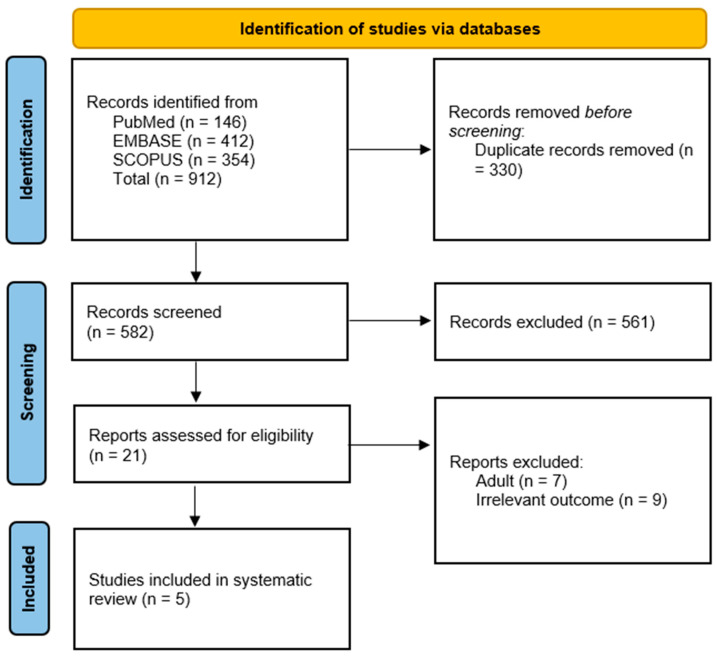
PRISMA flow chart.

A total of 912 records were identified from PubMed (146), EMBASE (412), and SCOPUS (354). After removing 330 duplicates, 582 records underwent title and abstract screening, with 561 exclusions. The remaining 21 reports were assessed for full-text eligibility, leading to the exclusion of seven adult studies and nine with irrelevant outcomes. Ultimately, five studies met the inclusion criteria and were included in the systematic review [15,16,27,28,29], Figure 1.

### 3.1. Baseline Characteristics and Summary of the Included Studies

The included studies span multiple countries, including the Netherlands, the USA, Denmark, and China. Among them, three utilized a cross-sectional design [15,27,29], one was a retrospective cohort study [28], and one followed a prospective observational methodology [16]. The sample sizes range from 37 to 92 participants, with most studies including a higher proportion of female patients. Age at surgery varies across studies, with median or mean ages between approximately 13.7 and 15.5 years. The studies primarily focus on pediatric and adolescent patients diagnosed with either DTC or Graves’ disease. Follow-up periods range from a few months to several years, with one study reporting a median follow-up of 17.8 years. HRQoL was assessed using validated tools such as PedsQL, EORTC QLQ-C30, THYCA-QoL, and WHOQOL-100, examining various domains, including fatigue, anxiety, depression, and psychosocial well-being. The surgical interventions analyzed include total thyroidectomy, hemithyroidectomy, and bilateral or unilateral thyroidectomy. Further details are shown in Table 1.

### 3.2. Quality Assessment

Two studies (Nies et al., 2016, and Su et al., 2024) achieved the highest quality rating with scores of 9/9 [15,16]. The remaining studies were rated as moderate quality, with scores ranging from 5 to 6 [27,28,29]. Notably, comparability scores were low in most studies, indicating potential limitations in adjusting for confounding variables (Table 2).

### 3.3. Qualitative Synthesis

#### 3.3.1. Emotional Domain (e.g., Anxiety, Depression, Emotional Resilience)

Thyroid surgery in children has substantial emotional and psychological effects. Stokhuijzen et al. (2015) reported that pediatric patients often experience low self-esteem and anxiety related to visible scarring from surgery. The CHQ-CF87 used in this study assessed various dimensions of emotional well-being, revealing that younger patients reported significantly lower scores in physical functioning compared to normative data [28]. Su et al. (2024) found that emotional function scores in the Pediatric Quality of Life Inventory (PedsQL) were higher at 1, 3, and 6 months, while total PedsQL scores were higher at 1 and 3 months, with no significant differences at 6 and 12 months [16]. Also, In EORTC QLQ-C30 outcomes, the unilateral thyroidectomy (UT) group had higher emotional function and global quality of life scores at 3, 6, and 12 months, but not at 1 month. Fatigue scores were lower in the UT group at 3, 6, and 12 months, while other functional scales and symptom scores showed no significant differences between groups [16]. Rasmussen et al. (2022) highlighted that total thyroidectomy improved disease-specific QoL, particularly reducing anxiety (median change, −33.33; *p* = 0.010) and emotional susceptibility (median change, −28.99; *p* = 0.035), suggesting a psychological benefit despite initial concerns over scarring [27]. Pérez et al. (2023) reported that thyroid cancer (TC) patients exhibited significantly lower overall, physical, psychosocial, emotional, and school functioning compared to healthy peers. Distress was a major predictor of lower HRQoL scores, emphasizing the need for psychological support for these patients [29].

#### 3.3.2. Social Domain (e.g., Peer Interaction, Scar Stigma, School Life)

Social functioning is another critical aspect of QoL that is affected by thyroid surgery in pediatric patients. Research by Su et al. (2024) indicated that children and adolescents who underwent bilateral thyroidectomy reported a lower health-related quality of life (HRQOL) compared to those who had unilateral procedures [16]. The visibility of scars and the associated stigma can lead to social withdrawal and avoidance of activities that were once enjoyable. Additionally, the study by Nies et al. (2016) found that survivors reported feeling less confident in social situations, which further exacerbated their feelings of isolation [15]. Rasmussen et al. (2022) found that families reported high satisfaction with surgical outcomes and minimal concerns over scar appearance, suggesting that while initial distress is common, long-term social adaptation may improve [27]. Despite this, social anxiety remains a significant barrier to reintegration, necessitating targeted interventions. Pérez et al. (2023) further reinforced this by reporting that thyroid cancer patients had significantly lower social functioning compared to healthy youth [29]. However, when compared to other cancer patients, thyroid cancer patients reported markedly better social functioning.

#### 3.3.3. Physical Domain (e.g., Fatigue, Activity Limitation, Somatic Symptoms)

Physical health and functioning are paramount in assessing QoL after thyroid surgery. Nies et al. (2016) found that while many patients reported overall normal QoL, specific physical complaints, such as fatigue and limitations in physical activities, were prevalent. The Multidimensional Fatigue Inventory (MFI-20) indicated that a significant proportion of survivors experienced fatigue that interfered with their daily activities [15]. The Short Form-36 (SF-36) results indicated that patients who underwent total thyroidectomy had lower scores in physical functioning compared to those who had hemithyroidectomy. For example, patients who underwent total thyroidectomy reported difficulties in performing physical activities such as sports or even routine tasks like lifting objects [15]. Stokhuijzen et al. (2015) further emphasized that the type of surgical procedure—total versus hemithyroidectomy—had implications for physical recovery, with total thyroidectomy patients reporting more significant physical limitations [28]. Rasmussen et al. (2022) reported significant improvements in physical and school-related functioning following total thyroidectomy [27]. Symptoms related to goiter, hyperthyroidism, fatigue, and cognitive impairment also showed postoperative improvement. Additionally, Graves’-disease-associated thyroid eye disease, which had low scores at baseline, improved after surgery. Pérez et al. (2023) supported these findings, reporting that thyroid cancer patients had a significantly lower physical HRQoL compared to healthy youth [29]. However, when compared to other cancer patients, thyroid cancer patients reported better physical functioning.

#### 3.3.4. Information Needs and Support Systems

The need for comprehensive information and support systems is critical for enhancing the QoL of pediatric thyroid-surgery patients. Rasmussen et al. (2022) reported that families generally felt well-supported during recovery, with most patients achieving full recovery within a median of 2 months [27]. Despite this, structured educational programs remain necessary to address knowledge gaps and improve long-term well-being. Similarly, Pérez et al. (2023) emphasized that caregiver perceptions of QoL aligned closely with patient-reported outcomes [29].

#### 3.3.5. Surgical Approaches and Their Impact on Quality of Life

Su et al. (2024) found that patients who underwent unilateral thyroidectomy reported higher HRQOL scores compared to those who had bilateral procedures. This difference was particularly evident in the domains of emotional and physical functioning, where the unilateral group experienced fewer complications and better overall satisfaction with their surgical outcomes [16]. The THYCA-QoL results indicated that patients who underwent unilateral procedures had significantly lower scores in neuromuscular and psychological symptoms compared to those who underwent bilateral procedures. Rasmussen et al. (2022) found that, despite initial postoperative challenges, total thyroidectomy resulted in substantial QoL improvements without permanent complications such as recurrent laryngeal nerve damage or hypoparathyroidism [27].

#### 3.3.6. Factors Affecting Quality of Life

The QoL in adult survivors of pediatric differentiated thyroid carcinoma (DTC) is influenced by a variety of factors, as highlighted in the study by Nies et al. (2016) [15]. Notably, male survivors reported significantly higher levels of reduced motivation and depression compared to their female counterparts. Employment status also played a crucial role; those who were unemployed exhibited lower scores on physical functioning and the Physical Component Summary (PCS) of the HRQOL. Additionally, increased general, physical, and total fatigue correlated with unemployment. The study further revealed that treatment with a higher cumulative dose of radioactive iodine (131-I) correlated with increased complaints of headaches (*p* = 0.006). Survivors with recurrent or persistent disease and those with greater thyroid-stimulating hormone (TSH) suppression reported increased concerns about neck scar visibility. Marital status, educational level, age at follow-up, tumor characteristics, surgical complications, and follow-up duration were not significantly associated with QoL outcomes. This includes HRQoL, fatigue, anxiety, depression, or thyroid-cancer-specific HRQoL. Pérez et al. (2023) found that age, time since surgery, disease severity, a history of thyroid autoimmune disease (e.g., Graves’ or Hashimoto’s disease), and exposure to radioactive iodine (RAI) treatment had no significant impact on HRQoL [29].

#### 3.3.7. Age-Specific Considerations

While most included studies did not conduct stratified analyses by age group, Stokhuijzen et al., 2015 directly compared QoL outcomes based on current age and found that patients under 18 reported significantly a lower physical QoL compared to older counterparts, suggesting a potential age effect on recovery perception [28]. Nies et al. (2016) further suggested that younger adult survivors recalled more scar-related concerns, whereas older adults focused more on voice-related symptoms, highlighting the evolving nature of QoL priorities with age [15]. These findings underscore the importance of considering developmental stage in future research and clinical support strategies.

## 4. Discussion

### 4.1. Summary of Our Findings

Our systematic review reveals several crucial findings regarding the quality of life (QoL) outcomes in pediatric patients following thyroid surgery. The analysis of five high-quality studies demonstrates that thyroid surgery impacts multiple dimensions of children’s well-being, with varying effects across different surgical approaches and patient populations. The emotional and psychological domain showed notable improvements post-surgery, particularly in anxiety reduction and emotional susceptibility, as evidenced by Rasmussen et al.’s findings of significant decreases in anxiety scores [27]. This improvement may be attributed to the resolution of pre-operative symptoms and concerns, although some patients continued to experience challenges related to surgical scarring and body image [30]. Physical functioning outcomes demonstrated a complex pattern, with total thyroidectomy patients generally reporting lower physical functioning scores compared to those who underwent hemithyroidectomy. This difference likely arises from the more extensive nature of total thyroidectomy and the subsequent need for lifetime hormone replacement therapy. The findings from Su et al. (2024) particularly highlighted that unilateral thyroidectomy patients experienced better HRQOL outcomes, suggesting that preserving partial thyroid function may offer advantages for certain patient populations [16]. Social functioning emerged as a significant concern, with studies consistently reporting lower social interaction scores compared to healthy peers. However, an interesting finding was that thyroid cancer patients demonstrated better social functioning compared to patients with other types of cancer, possibly due to the relatively better prognosis and less intensive treatment regimens associated with thyroid cancer [31]. The impact of various demographic and clinical factors on QoL outcomes revealed some unexpected patterns. Male survivors reported higher levels of reduced motivation and depression, contrary to general population trends. Employment status emerged as a crucial determinant of QoL, with unemployment correlating with lower physical functioning scores and increased fatigue levels. These findings suggest that the impact of thyroid surgery extends beyond immediate medical outcomes to influence broader life outcomes.

### 4.2. Comparing Our Results with Previous Similar Systematic Reviews

Our findings are similar to previous studies focusing on the adult population. Landry et al. 2022 investigated the health-related quality of life (HRQoL) in patients who underwent total thyroidectomy and lobectomy for differentiated thyroid carcinoma [32]. The research reveals that patients experience significant changes in their HRQoL following surgery, with various factors such as age, gender, and the type of surgical procedure impacting their recovery and overall well-being. Notably, younger patients and those undergoing lobectomy reported better HRQoL outcomes compared to older patients and those who had total thyroidectomy. Walshaw et al. 2022 focused into the long-term effects of thyroid cancer treatment on patients’ quality of life [33]. It highlights that even years after treatment, patients may continue to face psychological and physical challenges, emphasizing the need for ongoing support and monitoring. The study also points out that factors such as anxiety, depression, and fatigue can affect patients’ daily lives. Both studies underscore the necessity for comprehensive care strategies that address not only the medical aspects of thyroid cancer treatment but also the psychosocial dimensions of recovery. 

### 4.3. Implications of Our Findings

When viewed through the lens of developmental psychology, our findings align with Erik Erikson’s psychosocial development theory, particularly regarding identity formation during adolescence [34]. The timing of thyroid surgery is often associated with critical developmental periods, potentially explaining the impact on emotional and social functioning [35]. The implications of our findings are substantial for clinical practice and patient care. First, the results suggest that the choice of surgical approach should consider both medical necessity and potential impacts on QoL. Second, the identification of specific risk factors for poor QoL outcomes enables more targeted support interventions. Third, the findings emphasize the need for long-term follow-up care that extends beyond physical-health monitoring to include psychological and social support. Future research should prioritize the development and adoption of standardized QoL assessment protocols specific to pediatric thyroid surgery patients. This includes defining optimal timing for assessments, selecting age-appropriate and validated instruments, and ensuring consistency across studies to enable meaningful comparisons and pooled analyses. Establishing consensus guidelines will facilitate more reliable monitoring of recovery trajectories and the identification of vulnerable subgroups needing tailored interventions.

### 4.4. Strengths and Limitations

The primary strength of this review lies in its comprehensive approach to assessing QoL outcomes across multiple domains. The inclusion of validated assessment tools and high-quality studies enhances the reliability of our findings. Additionally, in some included studies, longer follow-up periods offer preliminary insights into potential long-term implications of pediatric thyroid surgery. However, several limitations warrant consideration. The relatively small number of included studies (n = 5) reflects the limited research focus on pediatric populations in this field. The heterogeneity in assessment tools and outcome measures complicated direct comparisons between studies. Furthermore, the lack of standardized timing for QoL assessments may have influenced the reported outcomes. Cultural and healthcare system differences across study locations may also limit the generalizability of findings. Additionally, the included studies were limited by small sample sizes, which restrict statistical power and reduce the ability to perform subgroup analyses, particularly for age- or sex-specific outcomes. The heterogeneity in follow-up duration across studies—ranging from a few months to nearly two decades—further complicates cross-study comparisons. Such variability limits the ability to draw consistent conclusions about the pattern of quality-of-life recovery and may obscure time-sensitive changes in physical, emotional, or social functioning.

## 5. Conclusions

This systematic review provides compelling evidence that thyroid surgery can impact multiple aspects of pediatric patients’ quality of life. While surgical outcomes are generally positive, the complex interplay between physical, emotional, and social factors necessitates a comprehensive approach to post-operative care. Based on our findings, we recommend: (1) implementing standardized QoL assessments at regular intervals post-surgery; (2) developing age-appropriate psychological support programs; (3) establishing long-term follow-up protocols that address both medical and psychosocial needs; and (4) conducting larger, multicenter studies to further evaluate the impact of different surgical approaches on QoL outcomes.

## Figures and Tables

**Table 1:** Baseline characteristics and summary of the included studies.

ID	Participants	Age at Surgery	Gender Distribution	Marital Status	Employment Status	Country	Study Design	Inclusion Criteria	Primary Outcomes Related to QoL	Follow-up	Type of Surgery	Conclusion
Su et al. (2024) [16]	84 patients	Median age: 14.27 years	Not specified	Not specified	Not specified	China	Prospective observational study	Pediatric and adolescent patients with low-risk papillary thyroid carcinoma	HRQoL assessed using THYCA-QoL, PedsQL, EORTC QLQ-C30	1-, 3-, 6-, and 12-months post-surgery	Unilateral and bilateral thyroidectomy	Bilateral thyroidectomy is associated with lower HRQoL compared to unilateral thyroidectomy.
Pérez et al. (2023) [29]	92 youth with TC, 87 caregivers	15.47 years (SD = 2.53, range 8.56–23.36)	83.7% female	77.2% White non-Hispanic	Baseline, 12 months, and 24 months post-surgery	USA	Cross-sectional study	Pediatric thyroid cancer patients aged 8.5–23.4 years and their caregivers	HRQoL using PedsQL and Distress Thermometer	Baseline, 12 months, and 24 months post-surgery	Surgical intervention (total thyroidectomy)	Pediatric TC patients show resilience compared to other cancers but report lower HRQoL than healthy peers; early screening is essential.
Rasmussen et al. (2022) [27]	37 patient–parent pairs	Not specified	Not specified	Not specified	Not specified	Denmark	Cross-sectional study	Patients aged 12–19 undergoing total thyroidectomy for Graves’ disease, along with their parents, completed surveys before and at least six months post-surgery.	Disease-specific QoL	Not specified	Surgical intervention (total thyroidectomy)	In high-volume surgical settings with low complication rates, total thyroidectomy for pediatric Graves’ disease improves disease-specific QoL and psychosocial functioning with minimal scar-related concerns.
Nies et al. (2016) [15]	67 survivors, 56 controls	Not specified	86.6% female (survivors)	64.2% in a relationship	91.0% employed/full-time students	Netherlands	Cross-sectional study	Adult survivors of pediatric DTC diagnosed <18 years, follow-up ≥5 years	Generic HRQoL, fatigue, anxiety, depression, thyroid cancer-specific HRQoL	Median 17.8 years (range 5.0–44.7)	Total thyroidectomy and 131-I administration	Overall normal QoL in survivors, with mild impairments in some domains.
Stokhuijzen et al. (2015) [28]	40 patients	Mean age: 13.7 years	72.5% female	Not specified	Not specified	Netherlands	Retrospective cohort study	Patients who underwent thyroid surgery before age 19 years (2000–2012)	QoL assessed using CHQ-CF87 and WHOQOL-100	Not specified	Total thyroidectomy and hemithyroidectomy	QoL significantly affected by surgery; improved with age; hemithyroidectomy has fewer negative effects.

DTC: Differentiated Thyroid Carcinoma; HRQoL: Health-Related Quality of Life; PedsQL: Pediatric Quality of Life Inventory; EORTC QLQ-C30: European Organization for Research and Treatment of Cancer Quality of Life Questionnaire; CHQ-CF87: Child Health Questionnaire—Child Form 87; WHOQOL-100: World Health Organization Quality of Life Assessment; THYCA-QoL: Thyroid Cancer-Specific Quality of Life Questionnaire; 131-I: Radioactive Iodine Therapy.

**Table 2:** Quality assessment using the NOS tool.

ID	Study Design	Selection	Comparability	Outcome	Total Score/9	Quality
Su et al. (2024) [16]	Cohort	4	2	3	9	High quality
Pérez et al. (2023) [29]	Cross-sectional	4	0	2	6	Moderate quality
Rasmussen et al. (2022) [27]	Cross-sectional	3	0	2	5	Moderate quality
Nies et al. (2016) [15]	Cross-sectional	5	1	3	9	High quality
Stokhuijzen et al. (2015) [28]	Cohort	4	0	2	6	Moderate quality

## Data Availability

No new data were created or analyzed in this study. Data sharing is not applicable to this article.

## Abbreviations

The following abbreviations are used in this manuscript:

QoL	Quality of Life
DTC	Differentiated Thyroid Carcinoma
HRQoL	Health-Related Quality of Life
PedsQL	Pediatric Quality of Life Inventory
EORTC QLQ-C30	European Organization for Research and Treatment of Cancer Quality of Life Questionnaire-Core 30
THYCA-QoL	Thyroid Cancer-Specific Quality of Life Questionnaire
WHOQOL-100	World Health Organization Quality of Life-100
CHQ-CF87	Child Health Questionnaire-Child Form 87
SF-36	Short Form-36 Health Survey
MFI-20	Multidimensional Fatigue Inventory-20
NOS	Newcastle-Ottawa Scale
UT	Unilateral Thyroidectomy
TC	Thyroid Cancer
RAI	Radioactive Iodine
TSH	Thyroid-Stimulating Hormone
PRISMA	Preferred Reporting Items for Systematic Reviews and Meta-Analyses
MeSH	Medical Subject Headings

## Appendix A

**Table A1:** Search strategy for each database.

Database	Search Strategy
PubMed	((“Thyroidectomy”[Mesh] OR “Thyroid Gland/surgery”[Mesh] OR “thyroid surgery” OR “thyroidectomy” OR “total thyroidectomy” OR “partial thyroidectomy” OR “thyroid lobectomy” OR “minimally invasive thyroid surgery” OR “robot-assisted thyroidectomy” OR “endoscopic thyroid surgery” OR “thyroid resection” OR “thyroid gland removal”)) AND ((“Child”[Mesh] OR “Adolescent”[Mesh] OR “Pediatric”[Mesh] OR child* OR pediatric* OR adolescent* OR youth* OR “young patient*” OR “infant*” OR “teenager*” OR “neonate*” OR “newborn*”)) AND ((“Quality of Life”[Mesh] OR “QoL” OR “life quality” OR “health-related quality of life” OR “HRQoL” OR “psychosocial well-being” OR “psychosocial impact” OR “emotional well-being” OR “functional outcome*” OR “postoperative quality of life” OR “patient-reported outcomes” OR “PROs” OR “self-reported health status” OR “long-term outcomes” OR “health perception” OR “social functioning” OR “mental health” OR “psychological impact” OR “physical health”))
Scopus	TITLE-ABS-KEY(“thyroid surgery” OR thyroidectomy OR “total thyroidectomy” OR “partial thyroidectomy” OR “thyroid lobectomy” OR “minimally invasive thyroid surgery” OR “robot-assisted thyroidectomy” OR “endoscopic thyroid surgery” OR “thyroid resection” OR “thyroid gland removal”) AND TITLE-ABS-KEY(child* OR pediatric* OR adolescent* OR youth* OR “young patient*” OR “infant*” OR “teenager*” OR “neonate*” OR “newborn*”) AND TITLE-ABS-KEY(“quality of life” OR “QoL” OR “life quality” OR “health-related quality of life” OR “HRQoL” OR “psychosocial well-being” OR “psychosocial impact” OR “emotional well-being” OR “functional outcome*” OR “postoperative quality of life” OR “patient-reported outcomes” OR “PROs” OR “self-reported health status” OR “long-term outcomes” OR “health perception” OR “social functioning” OR “mental health” OR “psychological impact” OR “physical health”)
Embase	(‘thyroid surgery’/exp OR thyroidectomy OR ‘total thyroidectomy’ OR ‘partial thyroidectomy’ OR ‘thyroid lobectomy’ OR ‘minimally invasive thyroid surgery’ OR ‘robot-assisted thyroidectomy’ OR ‘endoscopic thyroid surgery’ OR ‘thyroid resection’ OR ‘thyroid gland removal’) AND (‘child’/exp OR ‘adolescent’/exp OR pediatric* OR child* OR youth* OR “young patient*” OR “infant*” OR “teenager*” OR “neonate*” OR “newborn*”) AND (‘quality of life’/exp OR ‘QoL’ OR ‘life quality’ OR ‘health-related quality of life’ OR ‘HRQoL’ OR ‘psychosocial well-being’ OR ‘psychosocial impact’ OR ‘emotional well-being’ OR ‘functional outcome*’ OR ‘postoperative quality of life’ OR ‘patient-reported outcomes’ OR ‘PROs’ OR ‘self-reported health status’ OR ‘long-term outcomes’ OR ‘health perception’ OR ‘social functioning’ OR ‘mental health’ OR ‘psychological impact’ OR ‘physical health’)
